# Normosmic Congenital Hypogonadotropic Hypogonadism Due to *TAC3/TACR3* Mutations: Characterization of Neuroendocrine Phenotypes and Novel Mutations

**DOI:** 10.1371/journal.pone.0025614

**Published:** 2011-10-21

**Authors:** Bruno Francou, Jérôme Bouligand, Adela Voican, Larbi Amazit, Séverine Trabado, Jérôme Fagart, Geri Meduri, Sylvie Brailly-Tabard, Philippe Chanson, Pierre Lecomte, Anne Guiochon-Mantel, Jacques Young

**Affiliations:** 1 Univ Paris-Sud, Faculté de Médecine Paris-Sud UMR-S693, Le Kremlin Bicêtre, France; 2 Assistance Publique-Hôpitaux de Paris, Hôpital Bicêtre, Service de Génétique Moléculaire, Pharmacogénétique et Hormonologie, Le Kremlin Bicêtre, France; 3 INSERM U693, IFR93, Le Kremlin-Bicêtre, France; 4 Universitatea de medicina si farmacie, Craiova, Romania; 5 Service d'Endocrinologie et des Maladies de la Reproduction and Centre de Référence des Maladies Endocriniennes Rares de la Croissance, Le Kremlin Bicêtre, France; 6 Service d'Endocrinologie, Hôpital Bretonneau, Tours, France; University of Córdoba, Spain

## Abstract

**Context:**

*TAC3/TACR3* mutations have been reported in normosmic congenital hypogonadotropic hypogonadism (nCHH) (OMIM #146110). In the absence of animal models, studies of human neuroendocrine phenotypes associated with neurokinin B and NK3R receptor dysfunction can help to decipher the pathophysiology of this signaling pathway.

**Objective:**

To evaluate the prevalence of *TAC3/TACR3* mutations, characterize novel *TACR3* mutations and to analyze neuroendocrine profiles in nCHH caused by deleterious *TAC3/TACR3* biallelic mutations.

**Results:**

From a cohort of 352 CHH, we selected 173 nCHH patients and identified nine patients carrying *TAC3* or *TACR3* variants (5.2%). We describe here 7 of these *TACR3* variants (1 frameshift and 2 nonsense deleterious mutations and 4 missense variants) found in 5 subjects. Modeling and functional studies of the latter demonstrated the deleterious consequence of one missense mutation (Tyr267Asn) probably caused by the misfolding of the mutated NK3R protein.

We found a statistically significant (p<0.0001) higher mean FSH/LH ratio in 11 nCHH patients with *TAC3/TACR3* biallelic mutations than in 47 nCHH patients with either biallelic mutations in *KISS1R*, *GNRHR*, or with no identified mutations and than in 50 Kallmann patients with mutations in *KAL1*, *FGFR1* or *PROK2*/*PROKR2*. Three patients with *TAC3/TACR3* biallelic mutations had an apulsatile LH profile but low-frequency alpha-subunit pulses. Pulsatile GnRH administration increased alpha-subunit pulsatile frequency and reduced the FSH/LH ratio.

**Conclusion:**

The gonadotropin axis dysfunction associated with nCHH due to *TAC3/TACR3* mutations is related to a low GnRH pulsatile frequency leading to a low frequency of alpha-subunit pulses and to an elevated FSH/LH ratio. This ratio might be useful for pre-screening nCHH patients for *TAC3/TACR3* mutations.

## Introduction

In the last two years, loss-of-function mutations in *TAC3* (MIM 162330) and *TACR3* (MIM 162332), the genes encoding neurokinin B (NKB) and its receptor NK3R, respectively, have been described in patients with non syndromic normosmic congenital hypogonadotropic hypogonadism (nCHH) (OMIM #146110), pointing to a fundamental role of this pathway in the physiology of the human gonadotrope axis [1]–[5]. The precise mechanisms by which these mutations cause gonadotropin deficiency and CHH are not yet clear [6], although we recently noted GnRH deficiency in such nCHH patients [3]. In rodents [7], sheep [8], [9], goat [10] and non human primates [11], NKB is expressed throughout the brain [12] and particularly by the same neurons that express kisspeptin and dynorphin. In rats, these neurons form a bilateral, interconnected network that projects to NK3R-expressing GnRH terminals in the median eminence [13]. Kisspeptins have a recognized effect on GnRH secretion [14]. In contrast, the neuroendocrine role of NKB is controversial, as agonists may have either inhibitory or excitatory effects, depending on the animal model and gender [11], [15]–[18]. In addition, *Tacr3* knock-out mice seem to be fertile [19] and the phenotype of the murine *Tac2* knock-out (ortholog of human *TAC3*) has not been yet reported. Detailed neuroendocrine studies of adult nCHH patients with *TAC3/TACR3* mutations may help to decipher the role of this signaling pathway in the pathophysiology of the gonadotrope axis.

The aims of the present work were 1) to evaluate the prevalence of variants in the *TAC3* and *TACR3* genes in our cohort of nCHH patients; 2) to characterize newly identified mutants responsible for nCHH, at the molecular and functional levels and 3) to examine the neuroendocrine profile of patients with biallelic *TAC3/TACR3* mutations to evaluate the hypothesis that NKB and its receptor participate in controlling the frequency of GnRH secretory pulses.

### Patients

From a cohort of 352 patients with CHH we selected 173 patients with normosmic nCHH and screened for *TAC3* and *TACR3* mutations. Gonadotropin deficiency in this setting is characterized by: 1) absent or incomplete puberty at age 18 years; 2) low plasma testosterone levels in men and low to low-normal estradiol levels in women plus low or normal serum gonadotropin levels; 3) otherwise normal pituitary function; 4) normal serum ferritin concentrations; 5) normal magnetic resonance imaging (MRI) of the hypothalamic-pituitary region; and 6) a normal sense of smell on olfactometry, and no anosmia/hyposmia in relatives [20].

### Neuroendocrine profiling of *TAC3/TACR3*-mutated nCHH patients

Since Topaloglu et al. first reported that *TAC3/TACR3* mutations can cause nCHH, studies of published pedigrees have shown that the transmission of this genetic form is autosomal recessive [1]–[4]. Thus, to study the pulsatility of gonadotropin and free alpha subunit secretion in nCHH patients harboring *TAC3/TACR3* mutations, we enrolled only patients with biallelic mutations, comprising 2 men partially described elsewhere [3] and a new female patient (see Case Reports). Free alpha subunit secretion was evaluated because it has been proposed as the best surrogate for GnRH secretion in nCHH patients with an apulsatile LH profile [21]–[23].

On the same way, for FSH/LH ratio studies, we included 11 patients with biallelic *TAC3/TACR3* mutations, of whom 6 are partially described elsewhere [3] and 5 are novel (see Case Reports). The FSH/LH ratios in these 11 patients were compared, using the same assays (see below), with those in patients with CHH of different genetic origins, namely 4 patients with biallelic mutations in *KISS1R*, 11 with biallelic mutations in *GNRHR*, 32 nCHH patients with no identified mutations in genes known to cause nCHH and also with those of 50 patients with Kallmann syndrome and mutations in *KAL1* (n = 19) or *FGFR1* (n = 17), or *PROK2*/*PROKR2* (n = 14) (Information S1).

## Results

### Case reports

**Figure 1:**
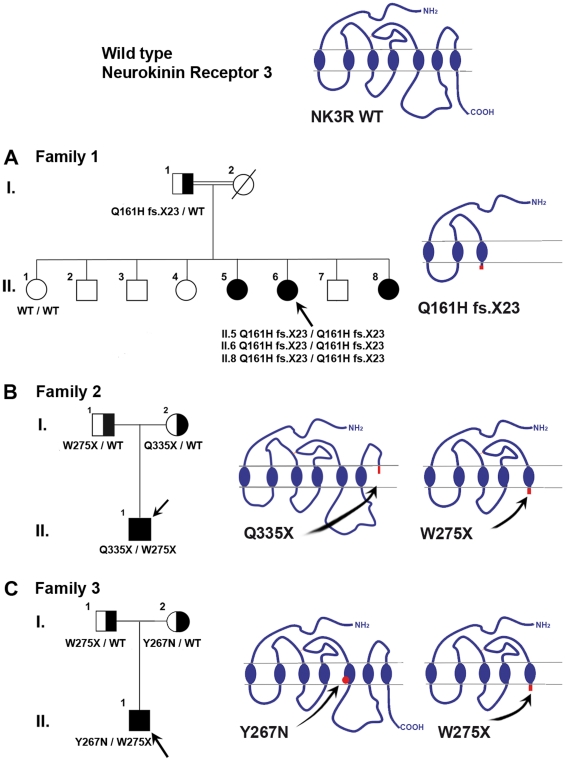
Family pedigrees and consequences of *TACR3* mutations on NK3R structure. Panel A. Pedigree of the family with homozygous *TACR3* c.483_499 deletion. The proband, subject II-6 (arrow), and her two affected sisters (subjects II-5 and II-8), were homozygous for the c.483_499 deletion. The unaffected father (I-1) was heterozygous for the mutation. The unaffected sister (II-1) carried homozygous wild-type alleles. This deletion results in the emergence of a premature stop codon (Q161HfsX23), truncating NK3R after the third transmembrane segment. Panel B. Pedigree of the family with compound heterozygous *TACR3* mutations c.824G>A and c.1003C>T. The proband, subject II-1 (arrow), was compound heterozygous for *TACR3* mutations c.824G>A and c.1003C>T. The unaffected father (I-1) was heterozygous for the c.824G>A mutation and the unaffected mother was heterozygous for the c.1003C>T mutation. The c.824G>A substitution produces a stop codon in the 5^th^ transmembrane segment (p.W275X) of NK3R. The c.1003C>T substitution produces a stop codon in the junction between the third extracellular loop and the seventh transmembrane domain (p.Q335X) of NK3R. Panel C. Pedigree of the family with compound heterozygous *TACR3* mutations c.799T>A and c.824G>A. The proband, subject II-1 (arrow), was compound heterozygous for *TACR3* mutations c.799T>A and c.824G>A. The unaffected father (I-1) was heterozygous for the recurent c.824G>A mutation and the unaffected mother was heterozygous for the c.799T>A mutation. This latter mutation affects a conserved amino acid in the fifth transmembrane domain (p.Y267N). Solid symbols indicate affected subjects and half-shaded symbols indicate unaffected heterozygotes. Circles represent female family members and squares male family members.

In **family 1,** three sisters were affected. The proband (subject II-6, Fig. 1A) was a young woman from a consanguineous family originating from Reunion Island. She was referred at age 24 years because pubertal development and menses had not occurred. She had typical signs of complete hypogonadism, with no breast development but the presence of pubic hair (P4). Her height was 164 cm, her weight 58 kg, and her bone age 14 years. Pelvic sonography showed a small uterus (34 mm high, 28 mm wide, 22 mm thick) and two small ovaries (right, 0.84 mL; left, 1.48 mL), with a few follicles less than 4 mm in diameter. Her karyotype was normal (46, XX). Combined estrogen-progestin replacement therapy induced breast development, an increase in uterus length, and regular menses. Hormonal evaluation after the end of this therapy showed that she still had very low estradiol and LH levels, an undetectable serum inhibin B level, but normal FSH levels as well as a nonpulsatile LH secretion, and an unchanged ovarian aspect on sonography. Pulsatile GnRH administration (90 ng/kg/pulse, every 90 minutes, subcutaneously) was started because she wished to conceive, resulting in increased circulating levels of LH, estradiol and inhibin B, appropriate endometrial thickening, and recruitment of a single dominant follicle of 20 mm. Eleven days later, while still receiving pulsatile GnRH therapy, her progesterone concentration rose to a luteal-phase level (19 ng/mL) and ultrasonography showed the typical aspect of a corpus luteum. This patient's older affected sister (subject II-5, Fig. 1A), who was evaluated at age 21 years, also had complete hypogonadism and a normal sense of smell. At diagnosis, she had no breast development and sparse pubic hair. Menarche had not occurred. The proband's younger affected sister (subject II-8, Fig. 1A) was 19 years old when first seen for primary amenorrhea and absent breast development. She was born at term with a normal birth weight after an uncomplicated pregnancy, and grew and developed normally until her early to mid-teen years. On examination, her height was 168 cm and her BMI 23 kg/m^2^. She was at Tanner breast stage 1, pubic hair stage 3, and axillary hair stage 2. Her bone age was 13.5 years at diagnosis.

At diagnosis, the 3 sisters had very low plasma estradiol levels (7.0, 10.7 and 8.6 pg/mL respectively; normal range in the early follicular phase: 22–90 pg/mL) and very low serum LH levels (0.05, 0.1, and 0.16 IU respectively; normal range 2.8–7.1 IU/L), while their FSH levels were normal for the early follicular phase (3.7, 4.3, and 4.6 IU/L respectively; normal range: 2.4–7.0 IU/L). Serum inhibin B levels were undetectable (<10 pg/mL) in the propositus and her older affected sister at diagnosis.

The **family 2** proband (subject II–1, Fig. 1B) was a Caucasian French man born to non consanguineous eugonadal French parents. He was referred at age 19 years-old for absent pubertal development. Physical examination showed typical signs of hypogonadism, with small testes (mean volume: 0.8 mL, normal range in men: 15–30 mL). His height was 180 cm and his weight 68 kg. His karyotype was normal (46, XY). His medical history indicated that he was first referred to a pediatric endocrinologist at age 8 years for micropenis (2.5 cm) and bilateral cryptorchidism, for which he was operated on at age 19 years. At diagnosis he had very low levels of plasma testosterone (0.2 ng/mL; normal range: 2.8–9.0 ng/mL) and serum LH (0.2 IU/L), but his serum FSH (5.4 IU/L) was normal for age and responded (peak: 8.0 IU/L) to GnRH challenge (100 µg intravenously). He was recently reevaluated in September 2010 at 46 years old after a four months interruption of androgen therapy: mean testicular volume was at 1 mL, serum testosterone (0.3 ng/mL) and serum inhibin B (12 pg/mL) were very low; serum LH was low (0.3 IU/L) and FSH was in the normal range (4.4 IU/L).

The **family 3** proband (subject II-1, Fig. 1C) was a Caucasian French man born to non consanguineous eugonadal French parents. He was referred at age 18 years for absent pubertal development. Physical examination showed typical signs of hypogonadism, with small intrascrotal testes (2 mL). His height was 183 cm and his weight 65 kg. His karyotype was normal (46, XY). He had very low levels of plasma testosterone (0.4 ng/mL) and serum LH (0.65 IU/L), but his serum FSH (5.6 IU/L) was normal for age and responded excessively (peak: 11.5 IU/L) to GnRH challenge. Interestingly, his medical history indicated that he was first referred to a pediatric endocrinologist at age 20 months for micropenis (2.3 cm) and right cryptorchidism, for which he was operated on at age 9 years. At age 20 months his levels of testosterone (0.1 ng/mL; normal range: 0.05–0.45) and LH (0.6 IU/L; normal range: 0.1–1.1 IU/L) were in the normal range for chronological age but the FSH was slightly elevated and responded excessively to GnRH challenge (from 2.2 (basal) to 11.2 IU/L (peak); normal range at 20 months: basal, 0.2–1.6 IU/L; peak, 2.3–5.9 IU/L) [24].

All the patients studied here had normal MRI of pituitary gland and olfactory bulbs, normal sense of smell on olfactometry and had no renal or craniofacial abnormalities, normal circulating iron, ferritin, and prolactin levels, and normal pituitary, adrenal and thyroid function. None had any other clear neurobehavioral or other phenotypic abnormalities (bimanual synkinesia, dysmorphic facial features, midline anomalies, hearing loss, hypotonia, ataxia, dementia, or polyneuropathy). In particular, all had normal learning ability.

### Molecular analysis

The *GNRH1, GNRHR, KISS1, KISS1R* and *FGFR1* exons and intron–exon boundaries were identical to the reference sequences in all the 173 patients studied. Of these 173 patients, 9 propositi (5.2%) carried *TAC3/TACR3* variants (1 in *TAC3* and 8 in *TACR3*) (Table 1 and Fig. S1). Two of these mutations, 1 in *TAC3* and 1 in *TACR3*, found in 4 propositi, were previously reported [3]. Here we describe 7 additional different *TACR3* variants (5 original and 2 recurrent) in 5 unrelated nCHH propositi (Table 1). Among them, 3 propositi carried biallelic *TACR3* mutations (see case reports), one propositus being homozygous (Fig. 1A), and two compound heterozygotes (Fig. 1B and 1C). In addition, we found 3 monoallelic *TACR3* variants in two nCHH propositi (Figs. S3 and S4).

**Table 1:** One *TAC3* and 8 *TACR3* * variants found in 9 propositi from a cohort of 173 normosmic CHH evaluated at Bicêtre Hospital.

Gene	Nucleotides change	Protein change	Fonctional consequences	Comment
*TACR3*	c.483_499del	Q161H fsX23	PTC/TP	novel
*TACR3*	c.689 G>A	R230H	no	novel
*TACR3*	c.738-1 G>A	Y247L fsX4	PTC/TP	reported in ref. 3
*TACR3*	c.799 T>A	Y267N	decrease MAPK activity	novel
*TACR3*	c.824 G>A	W275X	PTC/TP	recurrent in ref. 4
*TACR3*	c.857 A>G	K286R	no	recurrent (rs2276973)
*TACR3*	c.918 G>A	M306I	no	novel
*TACR3*	c.1003 C>T	Q335X	PTC/TP	novel
*TAC3*	c.209-1 G>C	P73I fsX9	PTC/TP	reported in ref. 3

PTC/TP: premature termination codon/truncated protein.

*see Fig. S1 in the supporting information.

In family 1 propositus, as well as in her affected sisters, we found a **homozygous** ***TACR3*** **deletion (c.483_499del)** (subjects II-6, II-5 and II-8, Fig. 1A and Fig. S2 panel A). The mutation was found in the heterozygous state in the patient's unaffected father (subject I-1, Fig. 1A and Fig. S2). The unaffected sister (subject II-1, Fig. 1A) was a wild-type homozygote (Fig. S2). This deletion results in a frameshift from codon 161 and the emergence of a premature stop codon at position 183 (Gln161HisfsX23), truncating NK3R after the third transmembrane segment (Fig. 1A and Fig. S1). This deletion was not found by genomic sequencing in 200 chromosomes from eugonadal ethnically matched subjects.

In propositus II.1 from family 2 (Fig. 1B) we found a **compound heterozygous** ***TACR3*** **mutation (c.824G>A and c.1003C>T).** The c.824G>A substitution produces a stop codon in the 5^th^ transmembrane segment (p.Trp275Stop) of NK3R (Fig. 1B, Fig. S1 and Fig. S2, panel B). This recurrent mutation has previously been reported in the homozygous and monoallelic states by Gianetti et al. [4]. It was found in the heterozygous state in the unaffected father. The c.1003C>T substitution which produces a stop codon in the junction between the third extracellular loop and the seventh transmembrane domain (p.Gln335Stop) of NK3R (Fig. 1B, Fig. S1) was found in the heterozygous state in the unaffected mother. These 2 mutations were not found by genomic sequencing in 200 chromosomes from ethnically matched eugonadal control subjects.

In propositus II.1 from family 3 (Fig. 1C) we found a **compound** **heterozygous** ***TACR3*** **mutation (c.799T>A and c.824G>A).** The p.Trp275stop recurrent mutation was found in the heterozygous state in the unaffected father (Fig. S2, panel C). The c.799T>A substitution, which produces a missense mutation (p.Tyr267Asn) located in the fifth transmembrane segment of NK3R (Fig. 1C), was found in the heterozygous state in the unaffected mother (Fig. S2, panel C). These 2 mutations were not found by genomic sequencing in 200 chromosomes from ethnically matched eugonadal control.

### Modeling and functional studies of the missense mutations

**Figure 2:**
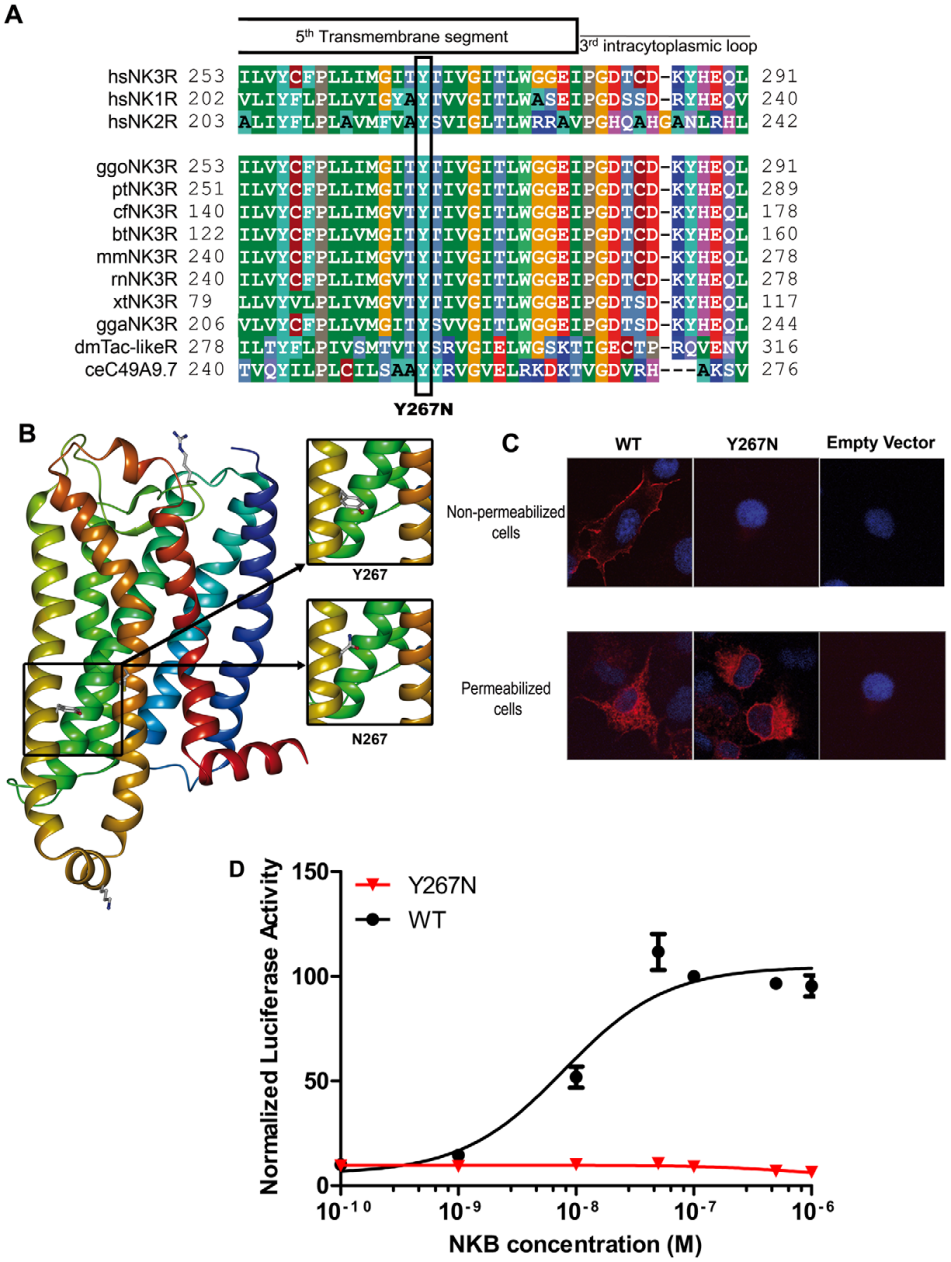
Molecular characterization, functional consequences and modeling of the p.Tyr267Asn *TACR3* mutation. Panel A. Evolutionary conservation of Tyr267. Tyr267 is perfectly conserved among NK3R orthologs and paralogs. The substitution is indicated below. Panel B. Modeling of the transmembrane region of NK3R. The tyrosine 267 and its substitution by an asparagine are pointed at the lipid bilayer. This position is extremely unfavorable for a polar residue such as asparagine. Panel C. Subcellular localization of ectopically expressed NK3R and Y267N mutant in non-permeabilized and permeabilized cells. Cells were transfected with the indicated expression vector and then treated for indirect immunofluorescence as described in the Methods section. The nuclei are counterstained by DAPI (blue). Upper panel: Z-stack projection of NK3R distribution in non-permeabilized cells obtained by confocal microscopy. Lower panel: fluorescence micrographs of fixed and permeabilized cells. Note the absence of Y267N NK3R mutant at the membrane (upper) despite its efficient expression in the cell (lower) whereas wild-type NK3R is localized at the plasma membrane. Panel D. NKB dose response of the reporter luc2P/SRE. Increasing concentrations of NKB led to an increase in the luciferase activity of wild-type NK3R (black circles). The mutant NK3R (red triangles) did not significantly enhance luciferase activity.

The Tyr267 residue is highly conserved among the three human tachykinin receptors and in all NK3R orthologs (Fig. 2A). Modeling was used to predict the potential impact of this point mutation on the three-dimensional organization of NK3R. We found that the hydrophobic Tyr267 residue is located in the middle of the fifth transmembrane segment and points towards the lipid bilayer (Fig. 2B). Introduction of an asparagine residue at position 267 (Y267N mutation) places a polar residue in a highly hydrophobic environment. Thus, it is likely that the asparagine side chain, in order to adopt a buried position, will induce transmembrane reorganization (twist or rotation). Therefore, the Tyr267Asn mutation leads probably to NK3R misfolding and dysfunction.

To test this misfolding hypothesis, we performed immunocytochemical studies in both permeabilized and non permeabilized cells in order to evaluate the subcellular localization of the Tyr267Asn mutant. In non permeabilized cells the wild-type NK3R molecule was located at the membrane (Fig. 2C, upper panel). In contrast, the Tyr267Asn mutant was not detected at the membrane, suggesting defective trafficking [25]. To ascertain that the mutant was efficiently expressed, we performed immunocytochemical experiments in permeabilized cells. In this condition, both wild-type and mutant proteins were detected. Compared to wild-type, the NK3R mutant was localized in the perinuclear region suggesting a misfolding (Fig. 2C, lower panel). Thus, the substitution of Tyr267 by asparagine impaired the proper targeting of the receptor to the cell surface.

To quantify and compare the stimulation effect of neurokinin B on wild-type and mutant NK3R, we performed dose-response curves for SRE luciferase assay in HEK 293T cells. Consistent with immunocytochemical and modeling studies, functional analyses clearly showed that mutated NK3R failed to stimulate a p44/42 MAP-sensitive SRE reporter gene contrary to the wild-type receptor which showed a clear dose-response stimulation in the presence of neurokinin B (Fig. 2D).

Similar modeling and functional studies were performed to characterize separately the 3 other variants (Lys286Arg, Met306Ile and Arg230His). In addition, the two Lys286Arg and Met306Ile mutations located on the same chromosome were assessed as double mutant. These analyses, detailed in supporting information (Fig. S3 and Fig. S4), suggest that in fact these variants did not affect the structure and the function of NK3R. Therefore they can not be considered as causative of the disease. For that reason, clinical features of these propositi were not detailed here.

### Analysis of free alpha subunit (FAS) and gonadotropin pulsatility in nCHH patients with biallelic *TAC3* or *TACR3* mutations

**Figure 3:**
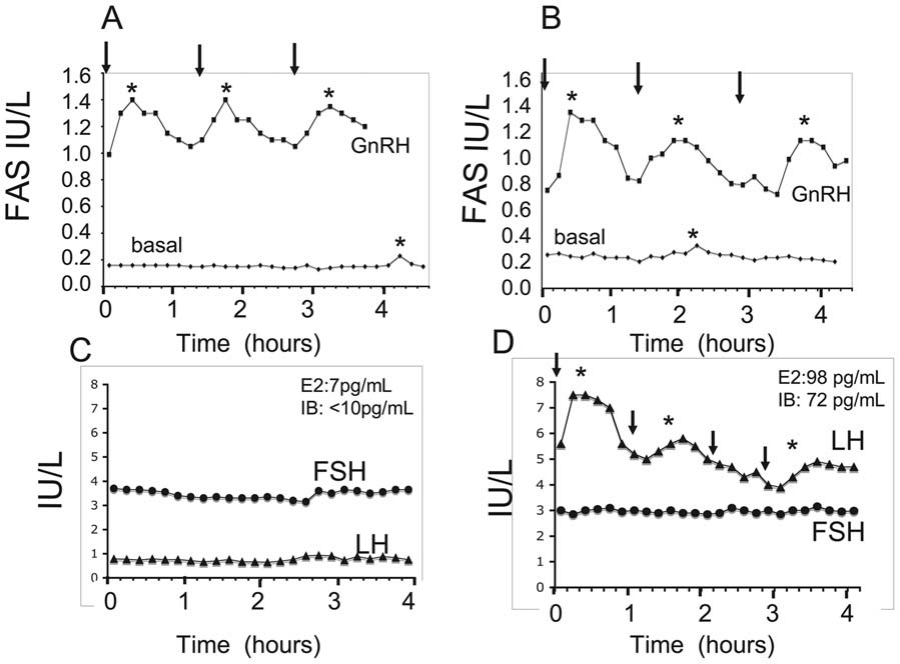
Secretory free alpha-subunit (FAS) and gonadotropin responses to pulsatile GnRH administration. Panel A. FAS concentrations in a man with nCHH caused by a homozygous *TAC3* c.209-1G>C mutation, before and on day 19 of pulsatile GnRH administration (patient reported in part in [3]). Arrows indicate exogenous GnRH boluses (7 µg). Panel B. FAS concentration in a woman with nCHH caused by a mutation in *TACR3* (subject II-6 in family I, Fig. 1A), before and on day 13 of pulsatile GnRH administration. Arrows indicate exogenous GnRH boluses (5 µg). Asterixes denote detectable FAS pulses, using Thomas' algorithm [24]. See *Patients* and *Methods* for details. Panel C. Pattern of gonadotropin secretion in a woman with complete nCHH and *TACR3* mutation (subject II-6 in family I, Fig. 1A). Basal LH concentration was very low and FSH concentration was in the normal range. Panel D. LH pulsatility in this woman was restored by pulsatile GnRH administration, and the serum FSH level fell slightly during pulsatile GnRH administration. Serum estradiol (E2) and inhibin B (IB) levels before and after GnRH administration are indicated respectively at the top of panel C and D.

Serum FAS pulsatility was analyzed in subject II.6 from family 1 (Fig. 1A and Table 2), who harbored a homozygous mutation in *TACR3*, and in two subjects partially described elsewhere [3] with either a homozygous *TAC3* or a homozygous *TACR3* mutation. Endogenous serum FAS levels are shown for two of these subjects in Fig. 3A and 3B.

**Table 2:** Characteristics of nCHH patients with biallelic *TACR3* or *TAC3* deleterious mutations selected for FSH/LH ratio calculation.

Cases	Age	FSH/LH Ratio	Sex	Gene	Nucleotides change	References
1*	24	74.0	Female	***TACR3***	c.[483_499del]+[483_499del]	Subject II-5 Family 1 present study
2*	21	43.0	Female	***TACR3***	c.[483_499del]+[483_499del]	Subject II-6 Family 1 present study
3*	19	28.7	Female	***TACR3***	c.[483_499del]+[483_499del]	Subject II-8 Family 1 present study
4	19	27.5	Male	***TACR3***	c.[824 G>A]+[1003 C>T]	Subject II-1 Family 2 present study
5	18	8.6	Male	***TACR3***	c.[799 T>A]+[824 G>A]	Subject II-1 Family 3 present study
6**	28	8.0	Male	***TACR3***	c.[738-1 G>A]+[738-1 G>A]	Subject II-1 Family 4 in ref. 3
7**	26	10.5	Female	***TACR3***	c.[738-1 G>A]+[738-1 G>A]	Subject II-5 Family 4 in ref. 3
8**	23	13.0	Female	***TACR3***	c.[738-1 G>A]+[738-1 G>A]	Subject II-7 Family 4 in ref. 3
9	31	32.0	Male	***TAC3***	c.[209-1 G>C]+[209-1 G>C]	Subject II-1 Family 1 in ref. 3
10	21	39.0	Male	***TAC3***	c.[209-1 G>C]+[209-1 G>C]	Subject II-1 Family 2 in ref. 3
11	26	6,6	Female	***TAC3***	c.[209-1 G>C]+[209-1 G>C]	Subject II-1 Family 3 in ref. 3

*members of the same kindred.

**members of the same kindred.

Mean (±SD) basal FAS concentrations in these 3 patients were low (0.19±0.05 IU/L), with a low mean pulse frequency (1.2±1.3 pulses every four hours (mean±SD); normal range: 2.3–3.0) and a low amplitude of detected pulses (0.2±0.1 IU/L). As expected, pulsatile GnRH administration to these 3 nCHH patients significantly (p<0.01) increased mean FAS levels (1.1±0.19 IU/L) and the FAS pulse frequency (3.3±0.6 pulses every four hours) and amplitude (0.41±0.11 IU/L, P<0.05), in line with GnRH dependency of pituitary FAS secretion [23].

Analysis of basal LH and FSH secretion overnight at 10-min intervals for 4 h in subject II.6 from family 1 (Fig. 1A and Table 2) showed very low levels and a nonpulsatile pattern of LH secretion, whereas mean baseline FSH levels were significantly higher (Fig. 3C). On day 13 of pulsatile GnRH administration (90 ng/kg/pulse, every 90 min, sc), pulses of LH were detected, synchronously with the GnRH boluses (Fig. 3D) and concomitantly with an increase in serum estradiol and inhibin B levels and a slight serum FSH decrease.

### Serum FSH/serum LH ratio in patients with *TAC3/TACR3* mutations

**Figure 4:**
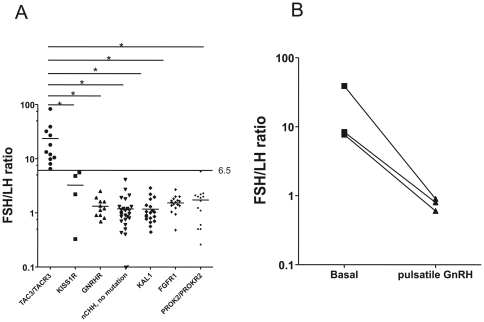
FSH/LH ratio in 11 patients with nCHH caused by biallelic *TAC3/TACR3* mutations. Panel A. Significantly higher serum FSH/ serum LH ratios in 11 patients with nCHH caused by *TAC3/TACR3* biallelic mutations than in patients with other genetic forms of CHH or in CHH patients with no mutation found in known genes. Note the Log scale on Y axis. A whole variance analysis by Kruskal-Wallis test (p<0.0001) was performed followed by post-hoc Newman-Keuls multiple comparison test; *indicates a significant difference between 2 groups (p<0.001). The threshold separating FSH/LH ratio in *TAC3/TACR3* mutated nCHH subjects from those of patients with other genetic forms of CHH is indicated by an horizontal line. Panel B. Decrease in the FSH/LH ratio in one patient with *TAC3* and two patients with *TACR3* mutations (see *Patients*) during pulsatile GnRH administration. Note the Log scale on Y axis.

The FSH/LH ratios in 11 subjects with biallelic *TAC3/TACR3* mutations (Table 2) are shown in Fig. 4A. Compared to subjects with other known genetic causes of nCHH *KISS1R (*mean *(*±*SD)* FSH/LH* = 3.2*±*2.4*), *GNRHR* (mean FSH/LH = 1.3±0.5) mutations or Kallmann syndrome (*KAL1:*mean FSH/LH = 1.2±0.6), *FGFR1* (mean FSH/LH = 1.5±0.5) and *PROK2* or *PROKR2* mutations (mean FSH/LH = 1.7±1.4)(Information S1) and to 32 patients with nCHH (mean FSH/LH = 1.2±0.8) and no identified genetic anomalies, patients with *TAC3/TACR3* mutations had very significantly (p<0.001 for each comparison, see Fig. 4A) higher FSH/LH ratios (mean FSH/LH =  23.6±22.4). Finally, we observed a decline in the FSH/LH ratio in the three subjects (patient II-6 family 1 and in the two nCHH subjects with respectively *TAC3* and *TACR3* mutations in part reported in ref 3) who received pulsatile GnRH administration (from 18.4±17.9 to 0.8±0.2)(Fig. 4B).

## Discussion

We found here *TAC3/TACR3* mutations in 5.2% of our nCHH population, a prevalence similar to that described by Gianetti et al [4] in a population of Caucasians, Asians and African-Americans. In this series, all the subjects with nCHH and biallelic *TAC3/TACR3* mutations were born to healthy heterozygous parents. This reinforces the autosomal recessive transmission of these two genetic forms of nCHH, as reported by Topaloglu et al. and us [1]–[3].

In the first family, we identified a new homozygous mutation in *TACR3* that leads to a frameshift and truncation of the receptor after the third transmembrane domain and therefore almost certainly has a deleterious effect. In two french families of Caucasian origin we found three *TACR3* mutations with compound heterozygous status. They share the same mutation, which generates a stop codon (Trp275stop) and leads to truncation of the protein after the fifth transmembrane domain, indicating that it is also certainly deleterious. This recurrent mutation has been found in the homozygous state in Caucasian American patients with nCHH [4], suggesting either a founder effect in this population or a mutation hotspot in this genomic region. The propositus of the second family also carried on the other allele a novel non sens mutation (Gln335stop) which leads to truncation of the protein after the third extra cellular loop also indicating that it is certainly deleterious. The propositus of the third family also carried a new missense mutation, Tyr267Asn, on the other allele, as revealed by studies of the parents. This mutation, affecting a conserved amino acid in the fifth transmembrane domain, affects the conformation of this domain, as shown by molecular modeling. The deleterious nature of this variant was confirmed by functional analyses showing altered MAP kinase-mediated NK3R signaling. This dysfunction is likely due to receptor misfolding and to defective plasma membrane targeting, as shown by immunocytochemical studies. Such a mechanism has already been reported for mutations affecting another 7-transmembrane-domain receptor (GnRHR) and leading to nCHH [25].

Analysis of gonadotropin concentrations in patients with biallelic *TAC3/TACR3* mutations showed very weak and apulsatile LH secretion, contrasting with preserved FSH concentrations. This profile, already found in other nCHH patients with biallelic *TAC3/TACR3* mutations [1], [3], points to the existence of low-frequency (and probably low amplitude) endogenous GnRH secretion [3]. We actually observed slow FAS secretion in all three patients studied, strongly supporting this hypothesis. Moreover, pulsatile GnRH administration at a physiological frequency re-established pulsatile LH and FAS secretion, and decreased the FSH/LH ratio. These pattern of response to pulsatile GnRH treatment are different from those reported in hypothalamic CHH patients, where GnRH leads to a rise in FSH, sometimes to supraphysiologic levels [26], further underlining the originality of the *TAC3/TACR3* mutated nCHH patients' neuroendocrine phenotype. All these data strongly suggest that the gonadotrope deficiency in subjects with *TAC3/TACR3* mutations is linked to a slowing of the frequency of endogenous GnRH secretion. It is therefore likely that neurokinin B, via its receptor NK3R, acts on the hypothalamus to regulate, either directly or indirectly (via kisspeptin/dynorphin/NKB neurons)[10], [13], the frequency of GnRH release into the hypothalamo-pituitary portal system. Our hypothesis is in line with a model recently proposed [10], [16] whereby kisspeptin, Dyn, and NKB act autosynaptically on kisspeptin neurons in the Arc to shape the pulsatile secretion of kisspeptin and hence GnRH release.

The molecular mechanisms by which low pattern of pulsatile GnRH secretion into portal blood favors FSH secretion by gonadotrope cells remain to be established. In contrast to the LHβ subunit gene, it has been shown in vitro that FSHβ subunit transcription is preferentially stimulated at low rather than high frequencies of pulsatile GnRH [27]. Because the synthesis of FSHβ is the rate-limiting step in FSH production, how FSHβ transcription is regulated is key to understanding the control of FSH release. In the context of the rFSHβ promoter, Ciconne et al. had recently established that GnRH stimulates transcription by increasing bound histone modifying enzyme CBP [28]. This increase in CBP is mediated in turn through the transcription factor CREB, bound to the FSH β CRE site. These authors also showed that mutation of this CRE site abolishes preferential FSH β transcription at low GnRH pulse frequency, implicating this site as an important mediator of GnRH pulse frequency-dependent FSH β gene expression [28].

The existence of a high FSH/LH ratio prior to any treatment in a significant number of patients with biallelic *TAC3/TACR3* mutations, contrary to patients with other genetic causes of CHH, suggests that this ratio could serve as a diagnostic marker to prescreen for *TAC3/TACR3* mutations in untreated patients with nCHH. This would narrow down the number of subjects in whom sequencing is necessary and would thus reduce the cost of genetic studies in this setting, although larger studies will be necessary before recommending this diagnostic approach.

All 11 patients with biallelic *TAC3/TACR3* mutations that we have analyzed to date had nCHH persisting into adulthood (from 18 yrs-old to 46 yrs-old). These results, in keeping with data reported by Topaloglu et al. [1], Guran et al. [2] and Fukami et al. [5], indicate that NKB and its receptor NK3R are crucial for physiological GnRH secretion after puberty, and not only during fetal life as suggested by Gianetti et al. [4]. Furthermore only one documented case of reversible nCHH in patients with *TAC3* biallelic mutations have been reported to date [4], indicating that this phenomenon does not predominate in this genetic form of CHH.

In conclusion, *TAC3/TACR3* mutations are an important genetic cause of nCHH that should be particularly searched in patients with a high serum FSH/LH ratio. Patients with nCHH and biallelic *TAC3/TACR3* mutations represent a useful model for deciphering the physiological role of NKB and its receptor NK3R in the gonadotrope axis. Such human models are all the more important as there is currently no genetic murine model with which to explore the precise consequences of this signaling dysfunction for hypothalamic GnRH secretion.

## Methods

All the participants gave their written informed consent for hormonal exploration and genetic analyses, in keeping with the provisions of the French Bioethics Law and the Declaration of Helsinki and after approval by the Bicêtre Hospital ethic committee (Comité de protection des personnes Ile de France, Hôpital Bicêtre).

### Hormone assays

We measured serum levels of LH, FSH, inhibin B, testosterone and estradiol levels with immunoradiometric assay, enzyme-linked immunosorbent assay, or radioimmunoassay, respectively, as previously reported [29], [30]. The detection limits of the LH and FSH assays were respectively 0.15 IU/L and 0.2 IU/L. The intra- and interassay coefficients of variation were, respectively, 1.5 and 5.2% for LH, 2.7 and 5.5% for FSH. Endogenous luteinizing hormone and alpha-subunit secretion, analyzed with Thomas' algorithm, was evaluated overnight at 10-minutes intervals, as reported elsewhere [29], [30], [31]. Serum free alpha-subunit (FAS) levels were measured as previously reported [32], using an immunoradiometric assay (IRMA) with two monoclonal antibodies (Immunotech, Marseille, France). Cross-reactivity of this immunoassay is less than 0.1% for dimeric hormones (including CG, LH, FSH, and TSH) and 0% for the free β-subunits of these hormones. Results are expressed as international units per liter (IU/L) of Medical Research Council 75/569, and the detection limit is 0.025 IU/L. Within-run and between-run coefficients of variation are respectively 6% and 12% at a concentration of 0.3 IU/L, and 3% and 5% at a concentration of 2 IU/L. Normal basal FAS levels are 0.9–1.9 IU/L for postmenopausal women, 0–0.6 IU/L for premenopausal women during the early follicular phase, and 0–0.3 IU/L for men.

For FSH/LH ratio determination, we used blood samples drawn at diagnosis from untreated CHH patients referred to the Endocrinology and Reproductive Diseases department of Hôpital Bicêtre and sera stored at −80°C. All gonadotropin assays were performed in a single run in order to avoid interassay variations.

### DNA analysis

Genomic DNA was extracted from white blood cells by using standard procedures.

*TAC3* coding exons 2, 3, 4, 5 and 6 and intron-exon junctions and *TACR3* coding exons 1, 2, 3, 4 and 5 and intron-exon junctions were amplified by PCR and sequenced as previously described [1], [3]. Sequence variations were found on both strands and confirmed in a separate PCR.

*GNRHR, GNRH1, KISS1R, KISS1, KAL1, FGFR1* and *PROK2/PROR2* were also analyzed as previously described, with minor modifications [29]–[31], [33]–[36].

### Modeling studies

The model of NK3R was generated by homology, using as template the crystal structure of rhodopsin, a hepta-transmembrane protein, with the Modeller package (version 9.8) [37]. Loops differing in length between NK3R and rhodopsin were reconstructed using the structural proteins bank (rotamer library) included in the O package [38]. NK3R mutants were generated using the same package. Statistics, calculated with Procheck, showed that >98.6% of the residues in the Ramachandran plot are in the most favored or allowed regions and that side-chain stereo parameters are inside the range or better than the statistics derived from a set of crystal structures of at least 2.0 Å resolution. In addition, the PROSAII program gave a combined Z-score (Cβ and surface potentials) of −3.34, a value in the range of structured proteins. These results suggest that the protein is of good quality and suitable for analysis.

### Directed mutagenesis

NK3R mutants were reproduced by site-directed mutagenesis using the pcDNA3.1+ plasmid encoding human NK3R with an hemagglutinin (HA) tag localized on the extracellular NK3R N-terminal extremity (Missouri S&T cDNA Resource Center) with the QuickChange Stratagen II kit (Stratagene, La Jolla, CA). Clones were verified by sequencing.

### Luciferase reporter gene assays

Tachykinin G protein-coupled receptors signal through several second messenger pathways including phosphoinositide and MAP kinase pathways (ERK 1/2) [39]. We thus used the luc2P*/*SRE/Hygro plasmid (Promega) which can induce luciferase production in response to MAP kinase activation as a reporter gene system [40]. HEK 293T cells (12,000 cells/well) were seeded 72 h before testing in high-glucose Dulbecco's minimal essential medium (Invitrogen, Cergy Pontoise, France) containing 2 mM glutamine, 100 IU/mL penicillin, 100 µg/mL streptomycin, and 10% heat-inactivated fetal calf serum at 37°C in 96-well plates. Twenty-four hours before testing, the cells were cotransfected in serum free OptiMEM, using Lipofectamine 2000 as recommended by the manufacturer (Invitrogen) with the plasmids coding for the wild-type or NK3R mutants, the luc2P/SRE/Hygro plasmid (Promega) and pRSV-beta-galactosidase plasmid. On the test day, neurokinin B was added at seven dilutions (from 10^−10^ to 10^−6^ M) to the culture medium. After incubation for 5–6 h the cells were harvested and assayed for β-galactosidase and luciferase activities as previously described [41], using a luminometer (Victor, Perkin Elmer). To standardize the transfection efficiency, the relative light units obtained in the luciferase assay were divided by the optical density obtained in the β-galactosidase assay. Maximum activity was considered at 5x10^−7^ M NKB. Prism version 3.02 (GraphPad Software Inc., SanDiego, CA) was used for curve fitting from at least three independent experiments performed in triplicate. Results are expressed as mean±SEM.

### Immunocytochemistry

COS-7 cells were grown in 24-well plates containing glass coverslips (14-mm diameter). After transfection with the expression vector coding for HA-NK3R, cells were processed as previously described [42]. Briefly, cells were fixed with 4% paraformaldehyde and permeabilized for 10 min with a 0.2% solution of Triton X100 diluted in PBS. Cells were then incubated overnight at 4°C with an anti-HA antibody (clone 3F10, Roche Applied Science), followed by an anti-rat fluorochrome-coupled secondary antibody (Dylight 549, Jackson ImmunoResearch Laboratories) for 30 min. Nuclear counterstaining was performed with 0.5 µg/mL DAPI (4′,6′-diamidino-2-phenylindole). Fluorescent cells were observed with an Olympus Provis AX70. Images were acquired with Qcapture Pro version 5.1 (Q Imaging Inc.). For live cell staining, cells were incubated for 1 h with the anti-HA antibody before fixation with 2% paraformaldehyde and direct incubation with the Dylight 549 secondary antibody. After extensive washing with TBS-Tween (0.1%), cells were post-fixed with 2% paraformaldehyde and counterstained with DAPI, rinsed quickly in water, and then mounted on slides with ProLong Gold mounting medium (Molecular Probes). A Zeiss LSM-510 confocal scanning laser microscope (Carl Zeiss, Thornwood, NY) was used to acquire Z-series of focal planes using a Plan Apochromat 63 oil objective.

## Supporting Information

Figure S1 **Schematic representation of the human NK3R variants found in a cohort of 173 normosmic CHH.** The mutated residues are indicated by red circles (see also Table 1).
(DOC)

Figure S2 **Molecular characterization of biallelic variants.** DNA sequencing of the genomic region encompassing the mutation. In-frame amino acids are indicated above each sequence. Panel A: Molecular characterization of the *TACR3* c.483_499 deletion in family 1 (see Fig. 1A). The *TACR3* c.483_499 deletion (delimited by the red vertical line) leads to a frameshift from codon 161 which is responsible for the emergence of a premature stop codon at position 183. This frameshift mutation was homozygous in the affected female propositus (Subject II-6) and heterozygous in her unaffected father (Subject I.1). A wild type homozygous unaffected sister (subject II-1) is indicated in the upper part of the panel. This gene product is 182 amino acids long, compared to 465 aa for the full-length protein. Only 3 transmembrane domains are encoded, rather than the 7 transmembrane domains in the wild-type receptor. Panel B: Molecular characterization of compound heterozygous *TACR3* mutations (c.824G>A and c.1003C>T) in family 2 (see Fig. 1B). The c.824G>A substitution produces a stop codon in the fifth transmembrane domain (p.Trp275stop  =  W275X) of NK3R. It was found in the heterozygote state in the unaffected father. The c.1003C>T substitution produces a stop codon at the junction between the third extra cellular loop and the seventh transmembrane domain (p.Gln335stop  =  Q335X) of NK3R. This mutation was found at the heterozygote state in the unaffected mother. Panel C: Molecular characterization of compound heterozygous *TACR3* mutations (c.799T>A and c.824G>A) in family 3 (Fig. 1C). The c.824G>A substitution is the same as in patient II-1 family 2, Fig. 1C. It was found in the heterozygous state in the unaffected father. The c.799T>A substitution produces a missense mutation (p.Tyr267Asn =  Y267N) located in the fifth transmembrane domain of NK3R. This mutation was found in the heterozygous state in the unaffected mother.
(DOC)

Figure S3 **Molecular characterization, functional consequences and modeling of Lys286Arg and Met306Ile NK3R variants.** In propositus II.2 from family 4 (panel A and panel B), we found **two variants (c.857A>G and c.918G>A).** The same variants were found in the unaffected father's (panel A) showing that it was not a real biallelic form. Lys286 is not conserved in the three human tachykinin receptors but is highly conserved in all NK3R orthologs (panel C). Using the three-dimensional model we found that Lys286 (K286) is located in the third intracellular loop (panel D)(see also Fig. S1). We tested the activity of the mutant receptor on the SRE luc reporter gene (panel E (upper)) and showed that there was no meaningful difference between the p.Lys286Arg mutant (green squares) and the wild-type NK3R (black circles). The c.918G>A produces a missense mutation (p.Met306Ile) located in the sixth transmembrane segment of NK3R (Fig. S1). It is poorly conserved in the three human tachykinin receptors and NK3R orthologs (panel C). Using the three-dimensional model, we found that Met306 (M306) is located at the middle of the sixth transmembrane segment and fully surface exposed (panel D). We tested the activity of the p.Met306Ile variant on the SRE-Luc reporter gene system (panel E (middle)) and showed that there was no difference between the p.Met306Ile mutant (green triangles) and the wild-type NK3R (black circles). To verify that the p.Lys286Arg variant did not interfere with the function of the p.Met306Ile mutant we performed a double p.Lys286Arg+p.Met306Ile mutant. The activity of the double mutant on the SRE-Luc reporter gene system (orange triangles) was similar to that in the wild-type NK3R (black circles) (panel E (lower)).
(DOC)

Figure S4 **Molecular characterization, functional consequences and modeling of Arg230His NK3R variant.** In propositus II.1 from family 5 (Panels A and B) we found **one variant (c.689G>A) at the heterozygous state**. This variant, located in the second extracellular loop of NK3R, produces a missense mutation (p.Arg230His)(see also Fig. S1). It is partly conserved in the three human tachykinin receptors and almost completely conserved in NK3R orthologs (Panel C). Using the three-dimensional model, we found that Arg230 (R230) is located in the second extracellular loop and solvent-exposed (Panel D). We tested the activity of the mutant receptor on the SRE-Luc system (Panel E) and showed that the Arg230His mutation has no consequence on the receptor activity.
(DOC)

Information S1 **Mutations in patients with other genetic causes of CHH or Kallmann syndrome included for the analysis of serum FSH/LH ratio (** **Fig. 4A** **).**
(DOC)

## References

[1] Topaloglu AK, Reimann F, Guclu M, Yalin AS, Kotan LD (2009). TAC3 and TACR3 mutations in familial hypogonadotropic hypogonadism reveal a key role for Neurokinin B in the central control of reproduction.. Nat Genet.

[2] Guran T, Tolhurst G, Bereket A, Rocha N, Porter K (2009). Hypogonadotropic hypogonadism due to a novel missense mutation in the first extracellular loop of the neurokinin B receptor.. J Clin Endocrinol Metab.

[3] Young J, Bouligand J, Francou B, Raffin-Sanson ML, Gaillez S (2010). TAC3 and TACR3 defects cause hypothalamic congenital hypogonadotropic hypogonadism in humans.. J Clin Endocrinol Metab.

[4] Gianetti E, Tusset C, Noel SD, Au MG, Dwyer AA (2010). TAC3/TACR3 Mutations Reveal Preferential Activation of Gonadotropin-Releasing Hormone Release by Neurokinin B in Neonatal Life Followed by Reversal in Adulthood.. J Clin Endocrinol Metab.

[5] Fukami M, Maruyama T, Dateki S, Sato N, Yoshimura Y (2010). Hypothalamic Dysfunction in a Female with Isolated Hypogonadotropic Hypogonadism and Compound Heterozygous TACR3 Mutations and Clinical Manifestation in Her Heterozygous Mother.. Horm Res Paediatr.

[6] Latronico AC (2009). The neurokinin B pathway in human reproduction.. Nat Genet.

[7] Burke MC, Letts PA, Krajewski SJ, Rance NE (2006). Coexpression of dynorphin and neurokinin B immunoreactivity in the rat hypothalamus: Morphologic evidence of interrelated function within the arcuate nucleus.. J Comp Neurol.

[8] Amstalden M, Coolen LM, Hemmerle AM, Billings HJ, Connors JM (2010). Neurokinin 3 receptor immunoreactivity in the septal region, preoptic area and hypothalamus of the female sheep: colocalisation in neurokinin B cells of the arcuate nucleus but not in gonadotrophin-releasing hormone neurones.. J Neuroendocrinol.

[9] Goodman RL, Lehman MN, Smith JT, Coolen LM, de Oliveira CV (2007). Kisspeptin neurons in the arcuate nucleus of the ewe express both dynorphin A and neurokinin B.. Endocrinology.

[10] Wakabayashi Y, Nakada T, Murata K, Ohkura S, Mogi K (2010). Neurokinin B and dynorphin A in kisspeptin neurons of thearcuate nucleus participate in generation of periodic oscillation of neural activity driving pulsatile gonadotropin-releasing hormone secretion in the goat.. J Neurosci.

[11] Ramaswamy S, Seminara SB, Ali B, Ciofi P, Amin NA (2010). Neurokinin B stimulates GnRH release in the male monkey (Macaca mulatta) and is colocalized with kisspeptin in the arcuate nucleus.. Endocrinology.

[12] Duarte CR, Schutz B, Zimmer A (2006). Incongruent pattern of neurokinin B expression in rat and mouse brains.. Cell Tissue Res.

[13] Rance NE, Krajewski SJ, Smith MA, Cholanian M, Dacks PA (2010). Neurokinin B and the hypothalamic regulation of reproduction.. Brain Res.

[14] Roseweir AK, Kauffman AS, Smith JT, Guerriero KA, Morgan K (2009). Discovery of potent kisspeptin antagonists delineate physiological mechanisms of gonadotropin regulation.. J Neurosci.

[15] Sandoval-Guzman T, Rance NE (2004). Central injection of senktide, an NK3 receptor agonist, or neuropeptide Y inhibits LH secretion and induces different patterns of Fos expression in the rat hypothalamus.. Brain Res.

[16] Navarro VM, Gottsch ML, Chavkin C, Okamura H, Clifton DK (2009). Regulation of gonadotropin-releasing hormone secretion by kisspeptin/dynorphin/neurokinin B neurons in the arcuate nucleus of the mouse.. J Neurosci.

[17] Corander MP, Challis BG, Thompson EL, Jovanovic Z, Loraine Tung YC (2010). The effects of neurokinin B upon gonadotrophin release in male rodents.. J Neuroendocrinol.

[18] Navarro VM, Castellano JM, McConkey SM, Pineda R, Ruiz-Pino F (2010). Interactions between kisspeptin and neurokinin B, in the control of GnRH secretion in the female rat.. Am J Physiol Endocrinol Metba.

[19] Siuciak JA, McCarthy SA, Martin AN, Chapin DS, Stock J (2007). Disruption of the neurokinin-3 receptor (NK3) in mice leads to cognitive deficits.. Psychopharmacology (Berl).

[20] Brioude F, Bouligand J, Trabado S, Francou B, Salenave S (2010). Non-syndromic congenital hypogonadotropic hypogonadism: clinical presentation and genotype-phenotype relationships.. Eur J Endocrinol.

[21] Winters SJ, Troen P (1988). Alpha-subunit secretion in men with idiopathic hypogonadotropic hypogonadism.. J Clin Endocrinol Metab.

[22] Whitcomb RW, O'Dea LS, Finkelstein JS, Heavern DM, Crowley WF (1990). Utility of free alpha-subunit as an alternative neuroendocrine marker of gonadotropin-releasing hormone (GnRH) stimulation of the gonadotroph in the human: evidence from normal and GnRH-deficient men.. J Clin Endocrinol Metab.

[23] Crowley WF, Taylor AE, Martin KA, Whitcomb RC, Finkelstein JS, Lustbader JW PD, Ruddon RW (1994). Use of the free alpha subunit (FAS) of glycoprotein secreting hormones as a surrogate marker of GnRH secretion in the human.. Glycoprotein hormones: structure, function, and clinical implications.

[24] De Luca F, Mitchell V, Wasniewska M, Arrigo T, Messina MF (2008). Regulation of spermatogenesis in McCune-Albright syndrome: lessons from a 15-year follow-up.. Eur J Endocrinol.

[25] Conn PM, Janovick JA (2009). Trafficking and quality control of the gonadotropin releasing hormone receptor in health and disease.. Mol Cell Endocrinol.

[26] Pitteloud N, Hayes FJ, Dwyer A, Boepple PA, Lee H (2002). Predictors of outcome of long-term GnRH therapy in men with idiopathic hypogonadotropic hypogonadism.. J Clin Endocrinol Metab.

[27] Ciccone NA, Kaiser UB (2009). The biology of gonadotroph regulation.. Curr Opin Endocrinol Diabetes Obes.

[28] Ciccone NA, Xu S, Lacza CT, Carroll RS, Kaiser UB (2010). Frequency-dependent regulation of follicle-stimulating hormone beta by pulsatile gonadotropin-releasing hormone is mediated by functional antagonism of bZIP transcription factors.. Mol Cell Biol.

[29] Salenave S, Chanson P, Bry H, Pugeat M, Cabrol S (2008). Kallmann's syndrome: a comparison of the reproductive phenotypes in men carrying KAL1 and FGFR1/KAL2 mutations.. J Clin Endocrinol Metab.

[30] Bouligand J, Ghervan C, Tello JA, Brailly-Tabard S, Salenave S (2009). Isolated familial hypogonadotropic hypogonadism and a GNRH1 mutation.. N Engl J Med.

[31] de Roux N, Young J, Misrahi M, Genet R, Chanson P (1997). A family with hypogonadotropic hypogonadism and mutations in the gonadotropin-releasing hormone receptor.. N Engl J Med.

[32] Chanson P, Pantel J, Young J, Couzinet B, Bidart JM (1997). Free luteinizing-hormone beta-subunit in normal subjects and patients with pituitary adenomas.. J Clin Endocrinol Metab.

[33] Dode C, Levilliers J, Dupont JM, De Paepe A, Le Du N (2003). Loss-of-function mutations in FGFR1 cause autosomal dominant Kallmann syndrome.. Nat Genet.

[34] de Roux N, Genin E, Carel JC, Matsuda F, Chaussain JL (2003). Hypogonadotropic hypogonadism due to loss of function of the KiSS1-derived peptide receptor GPR54.. Proc Natl Acad Sci U S A.

[35] Dode C, Teixeira L, Levilliers J, Fouveaut C, Bouchard P (2006). Kallmann syndrome: mutations in the genes encoding prokineticin-2 and prokineticin receptor-2.. PLoS Genet.

[36] Luan X, Zhou Y, Wang W, Yu H, Li P (2007). Association study of the polymorphisms in the KISS1 gene with central precocious puberty in Chinese girls.. Eur J Endocrinol.

[37] Eswar N, Webb B, Marti-Renom MA, Madhusudhan MS, Eramian D (2006). Comparative protein structure modeling using Modeller.. Curr Protoc Bioinformatics.

[38] Jones TA, Zou JY, Cowan SW, Kjeldgaard M (1991). Improved methods for building protein models in electron density maps and the location of errors in these models.. Acta Crystallogr A.

[39] Schmidlin F, Roosterman D, Bunnett NW (2003). The third intracellular loop and carboxyl tail of neurokinin 1 and 3 receptors determine interactions with beta-arrestins.. Am J Physiol Cell Physiol.

[40] Goze C, Berge G, M'Kadmi C, Floquet N, Gagne D (2010). Involvement of tryptophan W276 and of two surrounding amino acid residues in the high constitutive activity of the ghrelin receptor GHS-R1a.. Eur J Pharmacol.

[41] Pascual-Le Tallec L, Kirsh O, Lecomte MC, Viengchareun S, Zennaro MC (2003). Protein inhibitor of activated signal transducer and activator of transcription 1 interacts with the N-terminal domain of mineralocorticoid receptor and represses its transcriptional activity: implication of small ubiquitin-related modifier 1 modification.. Mol Endocrinol.

[42] Amazit L, Pasini L, Szafran AT, Berno V, Wu RC (2007). Regulation of SRC-3 intercompartmental dynamics by estrogen receptor and phosphorylation.. Mol Cell Biol.

