# Supplementary material for: Normosmic Congenital Hypogonadotropic Hypogonadism Due to TAC3/TACR3 Mutations: Characterization of Neuroendocrine Phenotypes and Novel Mutations
Source: PLoS One. 2011 Oct 21;6(10):e25614. doi: 10.1371/journal.pone.0025614 (PMC3198730; doi:10.1371/journal.pone.0025614)

**Figure S1 Schematic representation of the human NK3R variants found in a cohort of 173 normosmic CHH. The mutated residues are indicated by red circles (see also Table 1).**


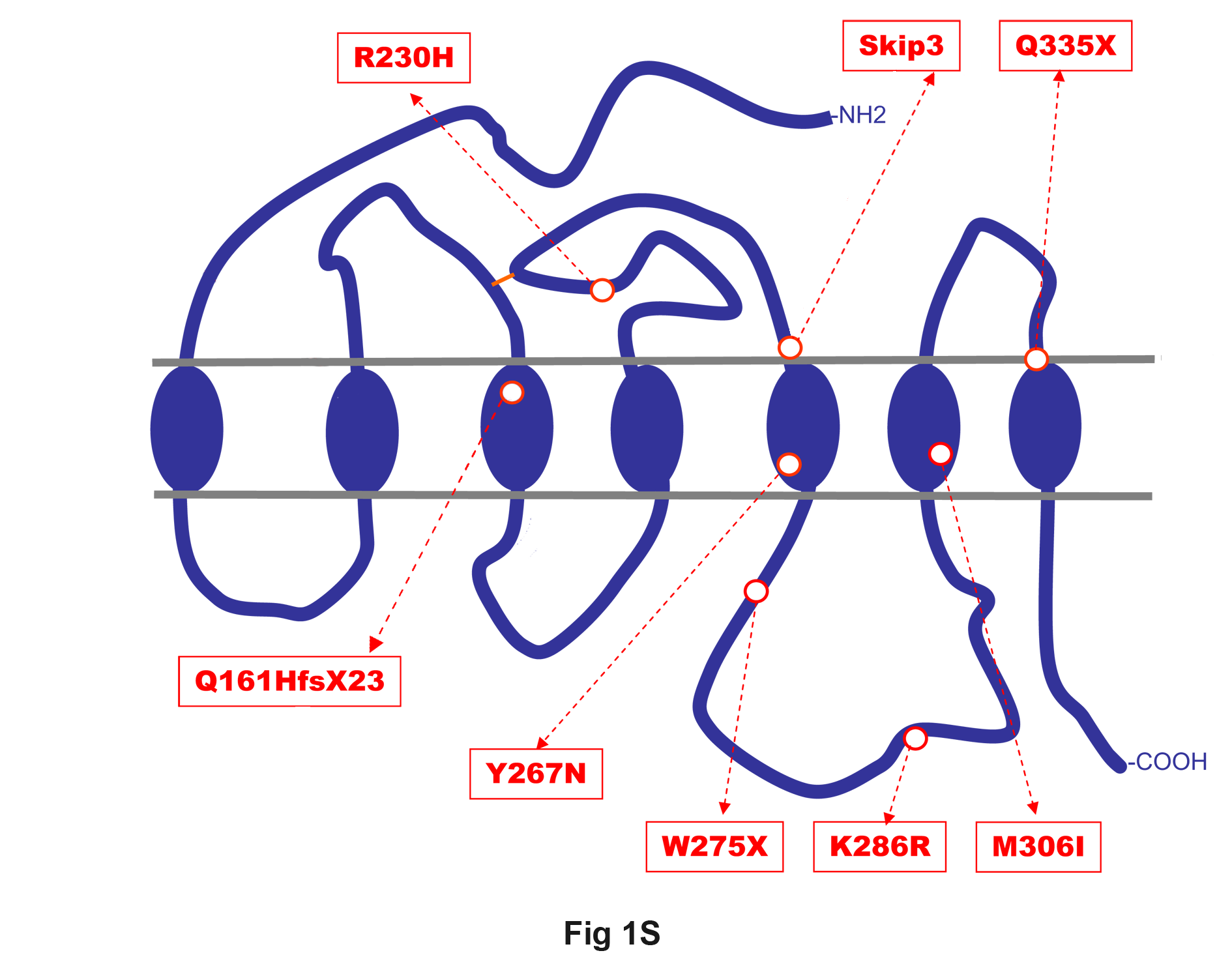

Supplement: Figure S1 — Schematic representation of the human NK3R variants found in a cohort of 173 normosmic CHH. The mutated residues are indicated by red circles (see also Table 1). (DOC) [file pone.0025614.s001.doc]
