# Supplementary material for: Normosmic Congenital Hypogonadotropic Hypogonadism Due to TAC3/TACR3 Mutations: Characterization of Neuroendocrine Phenotypes and Novel Mutations
Source: PLoS One. 2011 Oct 21;6(10):e25614. doi: 10.1371/journal.pone.0025614 (PMC3198730; doi:10.1371/journal.pone.0025614)
Supplement: Figure S2 — Molecular characterization of biallelic variants. DNA sequencing of the genomic region encompassing the mutation. In-frame amino acids are indicated above each sequence. Panel A: Molecular characterization of the TACR3 c.483_499 deletion in family 1 (see Fig. 1A). The TACR3 c.483_499 deletion (delimited by the red vertical line) leads to a frameshift from codon 161 which is responsible for the emergence of a premature stop codon at position 183. This frameshift mutation was homozygous in the affected female propositus (Subject II-6) and heterozygous in her unaffected father (Subject I.1). A wild type homozygous unaffected sister (subject II-1) is indicated in the upper part of the panel. This gene product is 182 amino acids long, compared to 465 aa for the full-length protein. Only 3 transmembrane domains are encoded, rather than the 7 transmembrane domains in the wild-type receptor. Panel B: Molecular characterization of compound heterozygous TACR3 mutations (c.824G>A and c.1003C>T) in family 2 (see Fig. 1B). The c.824G>A substitution produces a stop codon in the fifth transmembrane domain (p.Trp275stop = W275X) of NK3R. It was found in the heterozygote state in the unaffected father. The c.1003C>T substitution produces a stop codon at the junction between the third extra cellular loop and the seventh transmembrane domain (p.Gln335stop = Q335X) of NK3R. This mutation was found at the heterozygote state in the unaffected mother. Panel C: Molecular characterization of compound heterozygous TACR3 mutations (c.799T>A and c.824G>A) in family 3 (Fig. 1C). The c.824G>A substitution is the same as in patient II-1 family 2, Fig. 1C. It was found in the heterozygous state in the unaffected father. The c.799T>A substitution produces a missense mutation (p.Tyr267Asn = Y267N) located in the fifth transmembrane domain of NK3R. This mutation was found in the heterozygous state in the unaffected mother. (DOC) [file pone.0025614.s002.doc]

**Figure S2: Molecular characterization of biallelic variants.**
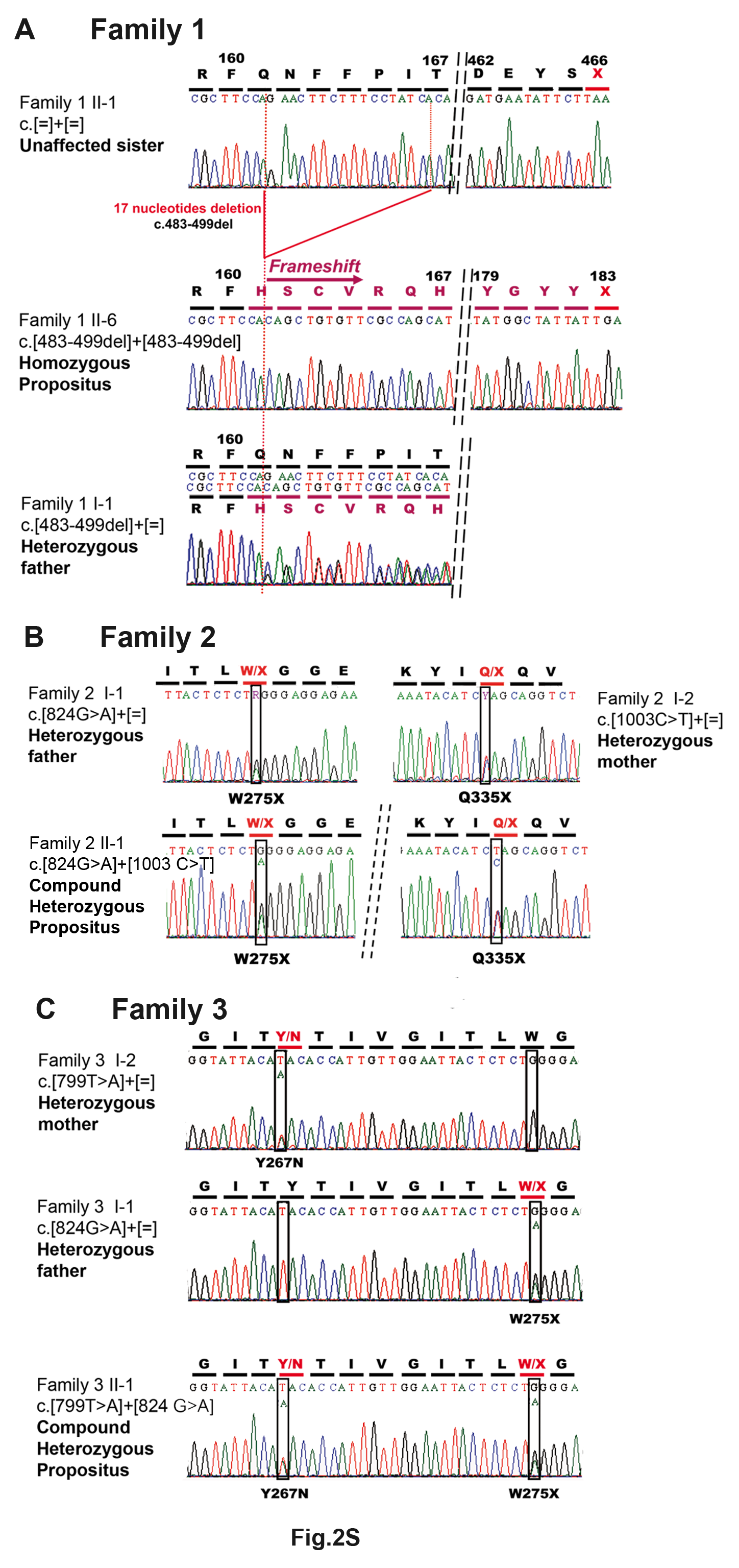


DNA sequencing of the genomic region encompassing the mutation. In-frame amino acids are indicated above each sequence.

**Panel A: Molecular characterization of the *TACR3* c.483_499 deletion in family 1 (see Fig. 1A).**

The *TACR3* c.483_499 deletion (delimited by the red vertical line) leads to a frameshift from codon 161 which is responsible for the emergence of a premature stop codon at position 183. This frameshift mutation was homozygous in the affected female propositus (Subject II-6) and heterozygous in her unaffected father (Subject I.1). A wild type homozygous unaffected sister (subject II-1) is indicated in the upper part of the panel. This gene product is 182 amino acids long, compared to 465 aa for the full-length protein. Only 3 transmembrane domains are encoded, rather than the 7 transmembrane domains in the wild-type receptor.

**Panel B: Molecular characterization of compound heterozygous *TACR3* mutations (c.824G>A and c.1003C>T) in family 2 (see Fig. 1B).** The c.824G>A substitution produces a stop codon in the fifth transmembrane domain (p.Trp275stop = W275X) of NK3R. It was found in the heterozygote state in the unaffected father. The c.1003C>T substitution produces a stop codon at the junction between the third extra cellular loop and the seventh transmembrane domain (p.Gln335stop = Q335X) of NK3R. This mutation was found at the heterozygote state in the unaffected mother.

**Panel C: Molecular characterization of compound heterozygous *TACR3* mutations (c.799T>A and c.824G>A) in family 3 (see Fig. 1C).** The c.824G>A substitution is the same as in patient II-1 family 2, Fig. 1C. It was found in the heterozygous state in the unaffected father. The c.799T>A substitution produces a missense mutation (p.Tyr267Asn= Y267N) located in the fifth transmembrane domain of NK3R. This mutation was found in the heterozygous state in the unaffected mother.
