# Supplementary material for: Normosmic Congenital Hypogonadotropic Hypogonadism Due to TAC3/TACR3 Mutations: Characterization of Neuroendocrine Phenotypes and Novel Mutations
Source: PLoS One. 2011 Oct 21;6(10):e25614. doi: 10.1371/journal.pone.0025614 (PMC3198730; doi:10.1371/journal.pone.0025614)
Supplement: Figure S3 — Molecular characterization, functional consequences and modeling of Lys286Arg and Met306Ile NK3R variants. In propositus II.2 from family 4 (panel A and panel B), we found two variants (c.857A>G and c.918G>A). The same variants were found in the unaffected father's (panel A) showing that it was not a real biallelic form. Lys286 is not conserved in the three human tachykinin receptors but is highly conserved in all NK3R orthologs (panel C). Using the three-dimensional model we found that Lys286 (K286) is located in the third intracellular loop (panel D)(see also Fig. S1). We tested the activity of the mutant receptor on the SRE luc reporter gene (panel E (upper)) and showed that there was no meaningful difference between the p.Lys286Arg mutant (green squares) and the wild-type NK3R (black circles). The c.918G>A produces a missense mutation (p.Met306Ile) located in the sixth transmembrane segment of NK3R (Fig. S1). It is poorly conserved in the three human tachykinin receptors and NK3R orthologs (panel C). Using the three-dimensional model, we found that Met306 (M306) is located at the middle of the sixth transmembrane segment and fully surface exposed (panel D). We tested the activity of the p.Met306Ile variant on the SRE-Luc reporter gene system (panel E (middle)) and showed that there was no difference between the p.Met306Ile mutant (green triangles) and the wild-type NK3R (black circles). To verify that the p.Lys286Arg variant did not interfere with the function of the p.Met306Ile mutant we performed a double p.Lys286Arg+p.Met306Ile mutant. The activity of the double mutant on the SRE-Luc reporter gene system (orange triangles) was similar to that in the wild-type NK3R (black circles) (panel E (lower)). (DOC) [file pone.0025614.s003.doc]

**Figure S3 :** **Molecular characterization, functional consequences and modeling of Lys286Arg and Met306Ile NK3R variants.**

**Figure S3 and S4 show three *TACR3* monoallelic variants found in two additional nCHH patients.** The 3 other *TACR3* variants in 2 patients from our cohort of 173 nCHH patients were: Arg230His, Lys286Arg and Met306Ile (see also Fig. 1S).


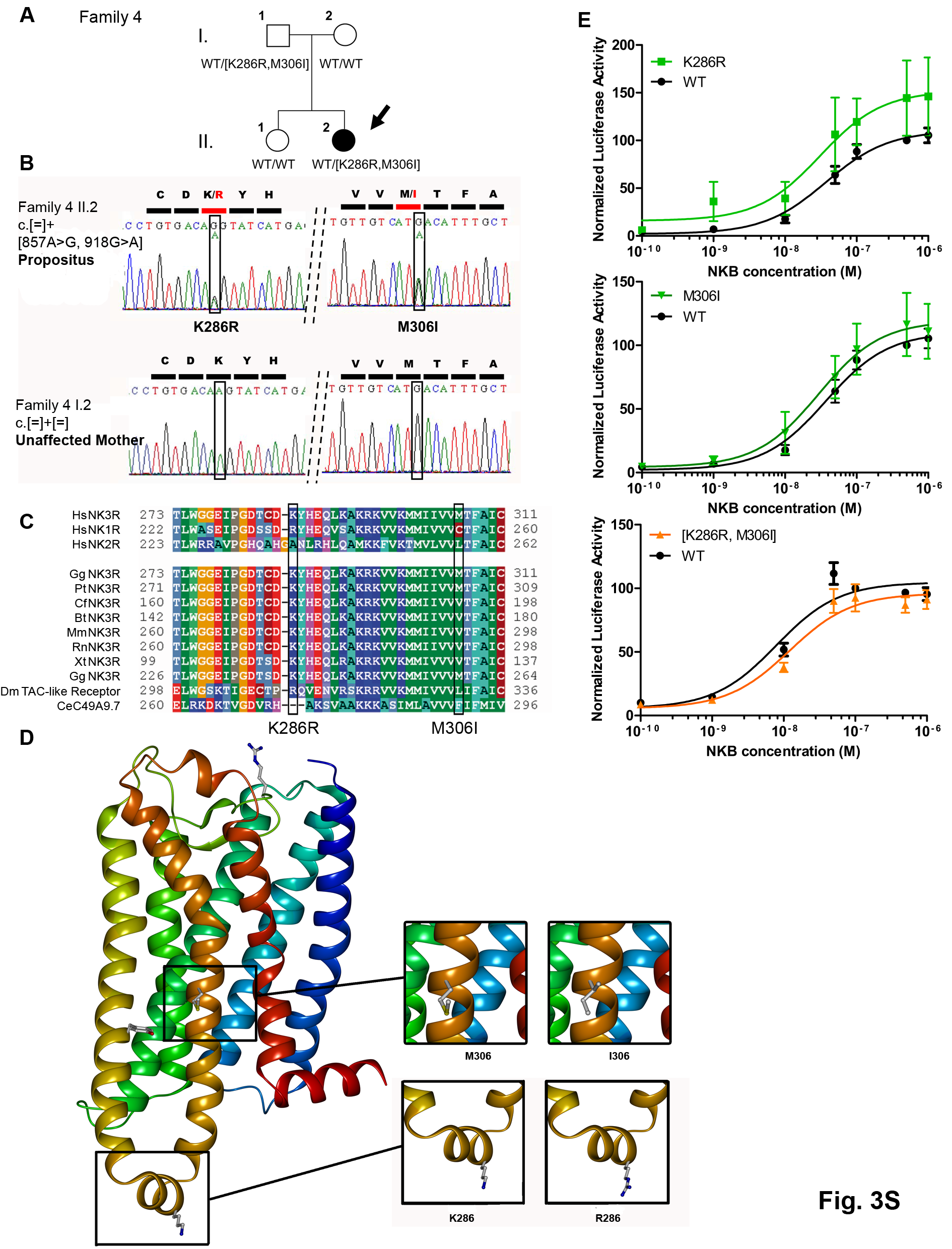


In propositus II.2 from family 4 (panel A and panel B), we found **two variants (c.857A>G and c.918G>A).** The same variants were found in the unaffected father’s (panel A) showing that it was not a real biallelic form.

The c.857A>G produces a missense mutation (p.Lys286Arg) and was previously described in SNP database (rs2276973). This polymorphism was found in 2 out of 200 chromosomes after sequencing of *TACR3* in eugonadal ethnically matched subjects. Lys286 is not conserved in the three human tachykinin receptors but is highly conserved in all NK3R orthologs (panel C). Using the three-dimensional model we found that Lys286 (K286) is located in the third intracellular loop (panel D)(see also Fig. 1S). This loop is involved in G coupled protein receptor transduction and interaction with beta arrestin (32). The positive charged residue Lys286 is solvent-exposed. Thus, it is likely that the introduction of an arginine residue at position 286 (R286), another basic amino acid, will not induce major structural changes (panel D). Moreover, according to sumoylation prediction software (SUMOsp 2.O), Lys286 does not seem to be engaged in post-translational modifications. We tested the activity of the mutant receptor on the SRE luc reporter gene (panel E (upper)) and showed that there was no meaningful difference between the p.Lys286Arg mutant (green squares) and the wild-type NK3R (black circles).

The c.918G>A produces a missense mutation (p.Met306Ile) located in the sixth transmembrane segment of NK3R (see Fig. 1S). This variant has not been previously described. We did not found it by sequencing 200 chromosomes of ethnically matched eugonadal subjects. It is poorly conserved in the three human tachykinin receptors and NK3R orthologs. Using the three-dimensional model, we found that Met306 (M306) is located at the middle of the sixth transmembrane segment and fully surface exposed. It forms van der Waals contacts with the hydrophobic part of the lipid bilayer. Introducing an isoleucine (I306), also a hydrophobic residue is likely to have only minor consequences on the three-dimensional organization of NK3R (panel D). We tested the activity of the p.Met306Ile variant on the SRE-Luc reporter gene system (panel E (middle)) and showed that there was no difference between the p.Met306Ile mutant (green triangles) and the wild-type NK3R (black circles). To verify that the p.Lys286Arg variant did not interfere with the function of the p.Met306Ile mutant we performed a double p.Lys286Arg+p.Met306Ile mutant. The activity of the double mutant on the SRE-Luc reporter gene system (orange triangles) was similar to that in the wild-type NK3R (black circles) (panel E (lower)).

Taken altogether, these results show that both variants do not impede the function of the receptor. Thus, the carrying patient has not been included in the neuroendocrine study.
