# Supplementary material for: Normosmic Congenital Hypogonadotropic Hypogonadism Due to TAC3/TACR3 Mutations: Characterization of Neuroendocrine Phenotypes and Novel Mutations
Source: PLoS One. 2011 Oct 21;6(10):e25614. doi: 10.1371/journal.pone.0025614 (PMC3198730; doi:10.1371/journal.pone.0025614)
Supplement: Figure S4 — Molecular characterization, functional consequences and modeling of Arg230His NK3R variant. In propositus II.1 from family 5 (Panels A and B) we found one variant (c.689G>A) at the heterozygous state. This variant, located in the second extracellular loop of NK3R, produces a missense mutation (p.Arg230His)(see also Fig. S1). It is partly conserved in the three human tachykinin receptors and almost completely conserved in NK3R orthologs (Panel C). Using the three-dimensional model, we found that Arg230 (R230) is located in the second extracellular loop and solvent-exposed (Panel D). We tested the activity of the mutant receptor on the SRE-Luc system (Panel E) and showed that the Arg230His mutation has no consequence on the receptor activity. (DOC) [file pone.0025614.s004.doc]

**Figure S4 :** **Molecular characterization, functional consequences and modeling of Arg230His NK3R variant.**


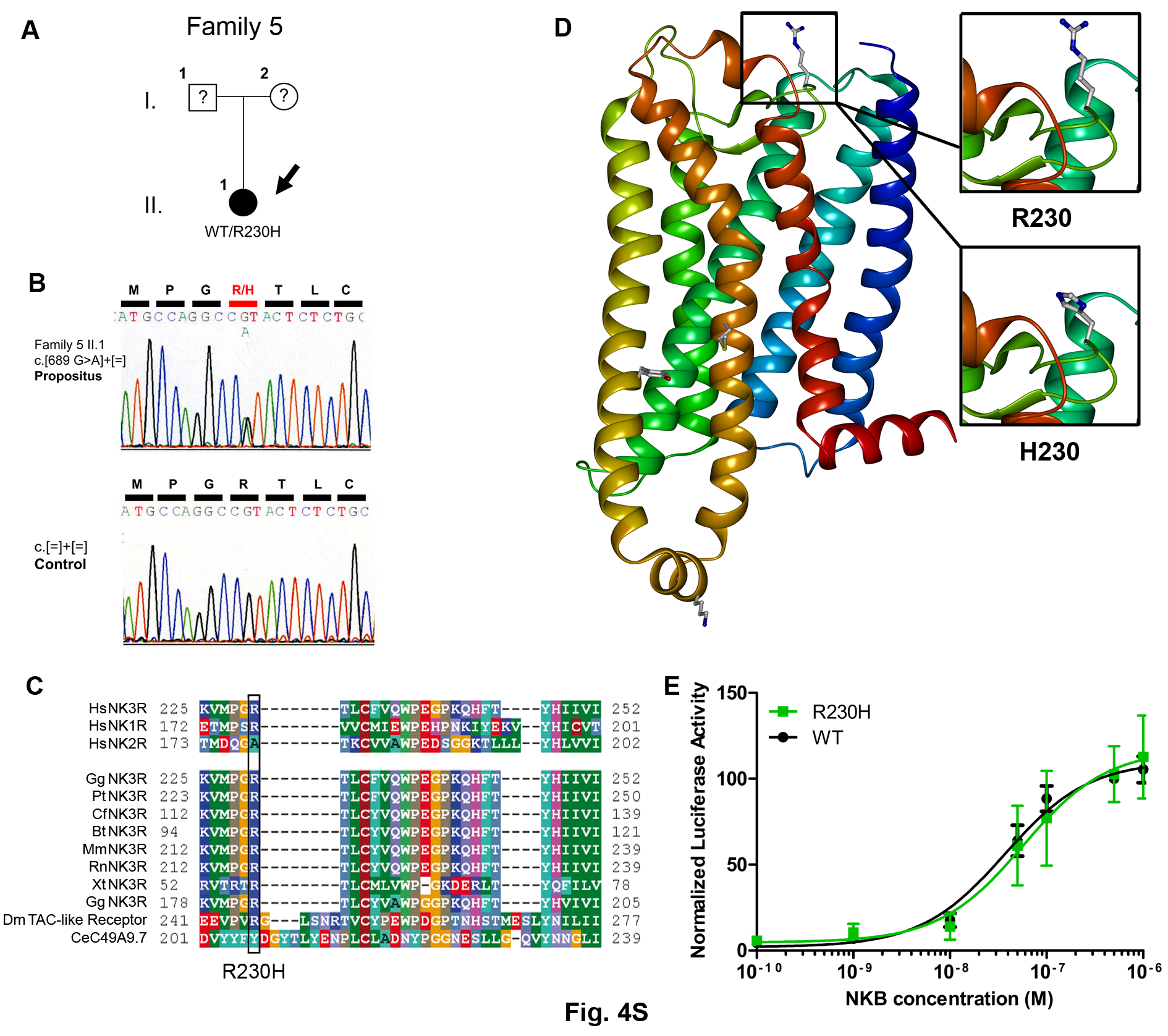


In propositus II.1 from family 5 (Panels A and B) we found **one variant (c.689G>A) at the heterozygous state**. This variant, located in the second extracellular loop of NK3R, produces a missense mutation (p.Arg230His)(see also Fig. 1S). It was not found by sequencing of 200 chromosomes of ethnically matched eugonadal subjects. It is partly conserved in the three human tachykinin receptors and almost completely conserved in NK3R orthologs (Panel C). Using the three-dimensional model, we found that Arg230 (R230) is located in the second extracellular loop and solvent-exposed (Panel D). Its substitution by another basic residue (H230) is likely to have only minor consequences on the three-dimensional organization of NK3R. We tested the activity of the mutant receptor on the SRE-Luc system (Panel E) and showed that the Arg230His mutation has no consequence on the receptor activity.

Thus, it is likely that this residue does not play an active role in the receptor function. The carrying patient has thus not been included in the neuroendocrine study.
