# Supplementary material for: Normosmic Congenital Hypogonadotropic Hypogonadism Due to TAC3/TACR3 Mutations: Characterization of Neuroendocrine Phenotypes and Novel Mutations
Source: PLoS One. 2011 Oct 21;6(10):e25614. doi: 10.1371/journal.pone.0025614 (PMC3198730; doi:10.1371/journal.pone.0025614)
Supplement: Information S1 — Mutations in patients with other genetic causes of CHH or Kallmann syndrome included for the analysis of serum FSH/LH ratio ( Fig. 4A ). (DOC) [file pone.0025614.s005.doc]

**Supplemental information 5**

**Mutations in patients with other genetic causes of CHH or Kallmann syndrome included for the analysis of serum FSH/LH ratio (see Fig.4A).**

**Biallelic *GPR54/KISS1R* loss of function mutations**

1- Female, 21 years: homozygous mutation: p.Leu148Ser; loss of function mutation

2- Female, 23 years: homozygous mutation: p.Leu148Ser

3- Male, 27 years: compound heterozygous p.Leu102Pro/Stop399Arg loss of function mutations

4- Male, 21 years: homozygous deletion of 155 bp lying between intron 4 and exon 5

**Biallelic *GnRHR* loss of function mutations**

1- Female, 24 years: homozygous c.869A>T; p.Tyr290Phe

2- Female, 26 years: homozygous c.869A>T; p.Tyr290Phe

3- Female, 22 years: homozygous c.806C>T; p.Thr269Met

4- Male 28 years: p.Pro282Arg/Tyr323Cys

5- Male 28 years: p.Leu266Arg/Leu266Arg

6- Male 19 years: p.Gln106Arg/Arg262Gln

7- Female 42 years: p.Gln106Arg/Arg262Gln

8- Male, 30 years: p.[Gln106Arg + Ser217Arg]/Arg262Gln

9- Female, 24 years: p.[Gln106Arg + Ser217Arg]/Arg262Gln

10- Female, 18 years: p.[Gln106Arg + Ser217Arg]/Arg262Gln

11- Female 30 years: p.Arg262Gln/Arg262Gln

**Homozygous *GnRH1* mutations**

1- Male 19 years: c.18-19insA/c.18-19insA

2- Female 17 years: c.18-19insA/c.18-19insA

**Hemizygous KAL1 mutations in male Kallmann patients**

1- 19 years: *Kal 1* deletion

2- 18 years: *Kal 1* deletion

3- 22 years: p.Cys90Gly

4- 17 years: p.Arg191fsX14

5- 20 years: p.Arg191fsX14

6- 30 years: c.769C>T, p.Arg257Stop

7- 18 years: p.Asn267Lys

8- 19 years: p.Asn267Lys

9- 21 years: p.Arg423Stop

10- 18 years: p.Arg423Stop

11- 21years: p.Arg424Stop

12- 26 years: IVS1+1G>A

13- 18 years: p.Glu552Stop

14- 26 years: p.Glu552Stop

15- 22 years: p.Val587Leu

16- 17 years: p.Pro551_Glu552delinsSerStop

17- 29 years: p.Pro551_Glu552delinsSerStop

18- 18 years: p.Gln57Stop

19- 21 years: p.Gln57Stop

**Heterozygous FGFR1 mutations in Kallmann patients**

1- Male, 18 years: p.Tyr99Cys

2- Female, 17 years: p.Ser139Stop

3- Female, 18 years: p.Ser139Stop

4- Female, 21 years: p.Ser139Stop

5- Female, 22 years: p.Ile300Thr

6- Male, 24 years: p.Lys321ArgfsX13

7- Male, 23 years: p.Thr340Met

8- Male, 20 years: p.Pro283Arg

9- Female, 24 years: p.Pro283Arg

10- Female 20 years: p.Pro366Leu

11- Male, 37 years: p.Val273Met

12- Male, 24 years: p.Val607Met

13- Male, 30 years: p.Val607Met

14- Female 20 years: p.Val607Met

15- Male 28 years: p.Glu670Lys

16- Male 25 years: p.Tyr730Stop

17- Male 23 years: p.Pro772Ser

***PROK2* or *PROKR2* mutations in Kallmann patients**

**PROK2**

1- Men 23 years: heterozygous c.94 G>C, p.Gly32Arg

2- Women 23 years: heterozygous c.297_299insT, p.Gly100fsX22+

3- Men 30 years: heterozygous c.297_299insT, p.Gly100fsX22/+

**PROKR2**

1- Men 18 years: heterozygous c.253C>T, p.Arg85Cys/+

2- Men 25 years: heterozygous c.253C>T, p.Arg85Cys/+

3- Men 30 years: heterozygous c.868C>T, p.Pro290Ser/+

4- Men 20 years: compound heterozygous c.802C>T/c.701G>A, p.Arg268Cys/Gly234Asp

5- Female 28 years: heterozygous c.253C>T, p.Arg85Cys

6- Men 22 years: homozygous c.518T>G/c.518T>G, p.Leu173Arg/Leu173Arg

7- Men 16 years: heterozygous c.802C>T, pArg268Cys/+

8- Men 20 years: heterozygous c.253C>G, p.Arg85Gly/+

9- Men 19 years: heterozygous c.518T>G, p.Leu173Arg/+

10- Men 41 years: heterozygous c.991G>A, p.Val331Met/+

11- Female 24 years: heterozygous c.518T>G, p.Leu173Arg/+
